# Implementation of virtual reality in healthcare: a scoping review on the implementation process of virtual reality in various healthcare settings

**DOI:** 10.1186/s43058-023-00442-2

**Published:** 2023-06-16

**Authors:** Marileen M. T. E. Kouijzer, Hanneke Kip, Yvonne H. A. Bouman, Saskia M. Kelders

**Affiliations:** 1 Centre for eHealth and Wellbeing Research; Department of Technology, Human & Institutional Behaviour, University of Twente, Enschede, Netherlands; 2 Department of Research, Transfore, Deventer, Netherlands

## Abstract

**Background:**

Virtual reality (VR) is increasingly used in healthcare settings as recent technological advancements create possibilities for diagnosis and treatment. VR is a technology that uses a headset to simulate a reality in which the user is immersed in a virtual environment, creating the impression that the user is physically present in this virtual space. Despite the potential added value of virtual reality technology in healthcare, its uptake in clinical practice is still in its infancy and challenges arise in the implementation of VR. Effective implementation could improve the adoption, uptake, and impact of VR. However, these implementation procedures still seem to be understudied in practice. This scoping review aimed to examine the current state of affairs in the implementation of VR technology in healthcare settings and to provide an overview of factors related to the implementation of VR.

**Methods:**

To give an overview of relevant literature, a scoping review was undertaken of articles published up until February 2022, guided by the methodological framework of Arksey and O’Malley (2005). The databases Scopus, PsycINFO, and Web of Science were systematically searched to identify records that highlighted the current state of affairs regarding the implementation of VR in healthcare settings. Information about each study was extracted using a structured data extraction form.

**Results:**

Of the 5523 records identified, 29 were included in this study. Most studies focused on barriers and facilitators to implementation, highlighting similar factors related to the behavior of adopters of VR and the practical resources the organization should arrange for. However, few studies focus on systematic implementation and on using a theoretical framework to guide implementation. Despite the recommendation of using a structured, multi-level implementation intervention to support the needs of all involved stakeholders, there was no link between the identified barriers and facilitators, and specific implementation objectives or suitable strategies to overcome these barriers in the included articles.

**Conclusion:**

To take the implementation of VR in healthcare to the next level, it is important to ensure that implementation is not studied in separate studies focusing on one element, e.g., healthcare provider-related barriers, as is common in current literature. Based on the results of this study, we recommend that the implementation of VR entails the entire process, from identifying barriers to developing and employing a coherent, multi-level implementation intervention with suitable strategies. This implementation process could be supported by implementation frameworks and ideally focus on behavior change of stakeholders such as healthcare providers, patients, and managers. This in turn might result in increased uptake and use of VR technologies that are of added value for healthcare practice.

Contributions to the literature

- Virtual reality is an innovative technology that is increasingly applied within different healthcare settings. Despite its potential to improve treatment, the adoption and uptake of VR are generally lacking.
- In this scoping review, we identified factors related to the implementation of VR that are important for successful adoption and effective use in practice. However, most often these factors are not sufficiently translated from research outcomes to healthcare practice.
- The findings of this scoping review contribute to the recognized gaps in the literature, stating recommendations for practice and future research on the systematic implementation of VR in healthcare.

## Background

Virtual reality (VR) is increasingly used in healthcare settings as recent technological advancements create possibilities for diagnosis and treatment. VR is a technology that uses a headset to simulate a reality in which the user is immersed in a virtual environment, creating the impression that the user is physically present in this virtual space [1, 2]. VR offers a broad range of possibilities in which the user can interact with a virtual environment or with virtual characters. Virtual characters, also known as avatars, can provide the user with a greater sense of reality and facilitate meaningful interaction [1]. VR interventions have been piloted in various healthcare settings, for example in treating chronic pain [3], improving balance in patients post-stroke [4], managing symptoms of depression [5], improving symptom burden in terminal cancer patients [6], and applied within treatment for forensic psychiatric patients [7]. These studies highlight the opportunities for VR as an innovative technology that could be of added value for healthcare. While there is a need for more research on the efficacy of VR in healthcare, experimental studies have shown that VR use is effective in improving the treatment of, among others, anxiety disorders [8], psychosis [9], or eating disorders [10]. However, the added value of VR is often not observed in practice due to the lack of usage of this technology.

Regarding uptake in clinical practice, VR is still in its infancy [11, 12]. Various barriers are identified as limiting the uptake, such as a lack of time and expertise on how to use VR in treatment, a lack of personalization of some VR applications to patient needs and treatment goals, or the gap in knowledge on the added value of VR in a specific setting [11, 13].

Not only VR uptake is challenging, but also other eHealth technologies experience similar difficulties in implementation [14]. eHealth is known as “the use of technology to improve health, well-being, and healthcare” [14]. For years, implementation has been out of scope for many eHealth research initiatives and healthcare practices, resulting in technologies that have not surpassed the level of development [15]. For these technologies to succeed and be used as effectively as intended, they must be well integrated into current healthcare practices and connected to the needs of patients and healthcare practitioners [13]. As a result, a focus on the implementation is of added value. It has the potential to improve the adoption, uptake, and impact of technology [16]. However, implementation procedures for VR technology still seem to be understudied in both research and practice [12, 17].

One of the reasons for the lacking uptake of (eHealth) technology is the complexity of the implementation process [18, 19]. The phase between the organizational decision to adopt an eHealth technology and the healthcare providers actually using the technology in their routine is complex and multifaceted [18, 19]. This highlights the importance of a systematic and structured implementation approach that fits identified barriers. The use of implementation strategies, known as the “concrete activities taken to make patients and healthcare providers start and maintain use of new evidence within the clinical setting,” can help this process by tackling the implementation barriers [20]. These strategies can be used as standalone, multifaceted, or as a combination [21]. Often, they are part of an implementation intervention, which describes what will be implemented, to whom, how, and when, with the strategies as a how-to description in the intervention [17]. In addition, according to Proctor et al. [22], it is important to conceptualize and evaluate implementation outcomes. Implementation outcomes, such as acceptability, adoption, appropriateness, feasibility, fidelity, implementation cost, penetration, and sustainability, can be used to set specific and measurable implementation objectives. Furthermore, assessing implementation outcomes will increase the understanding of the success of the implementation process and form a starting point for studies focusing on the effectiveness of VR in healthcare [22].

While implementation interventions could help the systematic implementation of VR, they are rarely used in practice. A way to stimulate systematic implementation and help develop an implementation intervention is by using an implementation model to guide this process. While a broad range of implementation models have been developed, there is still limited use of these models to structure the implementation of VR in healthcare [23]. One framework that could be used to identify important aspects of implementation is the NASSS framework, which investigates the *n*on-adoption, *a*bandonment, and challenges to *s*cale up, *s*pread, and *s*ustainability of technology-supported change efforts in health and social healthcare [24]. The NASSS framework does not only focus on the technology itself, but includes the condition of the target group, the value proposition, the adopter system (staff, patients, and healthcare providers), the healthcare organization(s), the wider system, and the embedding and adoption of technology over time [24]. The framework is used to understand the complexity of the adoption of new technologies within organizations [25]. However, it remains unclear if and what factors of the NASSS framework, or any other implementation framework, can be found in the implementation of VR in various healthcare settings.

In summary, virtual reality interventions have the potential to improve the quality of care, but only if implemented thoroughly. As VR use becomes more prevalent, studies should expand the focus to identify factors specifically related to the implementation of this new technology [19]. It is advised to perform a needs assessment, understand potential barriers to implementation early, set implementation objectives, and identify fitting implementation strategies before testing VR interventions in practice [26]. Therefore, this scoping review aims to examine the current state of affairs in the implementation of VR technology in healthcare settings and provide an overview of factors related to the implementation of VR. Within this research, the following sub-questions are formulated: (1) Which barriers play a role in the implementation of VR in healthcare? (2) Which facilitators play a role in the implementation of VR in healthcare? (3) What implementation strategies are used to implement VR in healthcare? (4) To what extent are specific implementation objectives and outcomes being formulated and achieved? (5) What are the recommendations for the implementation of VR in healthcare?

## Methods

To address the study aims, a scoping review was undertaken on the current state of affairs regarding the implementation of virtual reality in healthcare settings. Due to the broad scope of the research questions, a scoping review is most suitable to examine the breadth, depth, or comprehensiveness of evidence in a given field [23]. As a result, scoping reviews represent an appropriate methodology for reviewing literature in a field of interest that has not previously been comprehensively reviewed [24]. This scoping review is based on the methodological framework of Arksey and O’Malley [27] including the following steps: (1) identifying the research questions, (2) identifying relevant studies, (3) study selection, (4) charting the data, and (5) collating, summarizing and reporting the results. A protocol was developed and specified the research questions, study design, data collection procedures, and analysis plan. To the authors’ knowledge, no similar review had been published or was in development. This was confirmed by searching academic databases and the online platforms of organizations that register review protocols. The protocol was registered at OSF (Open Science Framework) under registration https://doi.org/10.17605/OSF.IO/5Z3MN. OSF is an online platform that enables researchers to plan, collect, analyze, and share their work to promote the integrity of research. This scoping review adheres to the Preferred Reporting Items for Systematic reviews and Meta-Analyses extension for Scoping Reviews (PRISMA-ScR) [26].

### Searches

A comprehensive, systematic electronic literature search was undertaken using three databases: Scopus, PsycINFO, and Web of Science. In each database, the same search strategy was used. Search terms were identified and included in the search strategy for three main categories relevant to the research questions: implementation, virtual reality, and healthcare. The search terms within a category were combined using the Boolean term “OR” and the term “AND”was used between the different categories. The search strategy was piloted to check if keywords and databases were adequate and adjustments were made whenever necessary. The full electronic search strategy can be found in Appendix 1.

### Study inclusion and exclusion criteria

All identified records published up until February 2022, that were peer-reviewed, and written in English, Dutch, or German, were included in the initial results. All references and citation details from different electronic databases were imported into the online review management system Covidence and duplicate records were removed automatically. A three-step screening approach, consisting of a title, abstract, and full-text screening, was used to select eligible studies.

Records were included if the titles indicated that the article focused on VR within a healthcare setting and that VR was used as a tool for prevention or treatment of patients. Because of the possibility of implementation not being mentioned in the title, broad criteria were used to prevent the unjust exclusion of relevant studies. In addition, records were included if they outline (parts of) the implementation process of VR technology (e.g., needs assessment, planning, execution, or lessons learned). Furthermore, the primary target group of the VR technology had to be patients with mental or physical disorders. If the studies focused solely on augmented reality (AR) or mixed reality (MR) and/or described a VR technology that was utilized to train healthcare professionals, they were excluded. Additionally, studies were excluded if full texts could not be obtained or if the study design resulted in no primary data collection, such as meta-analyses, viewpoint papers, or book chapters.

**Fig. 1:**
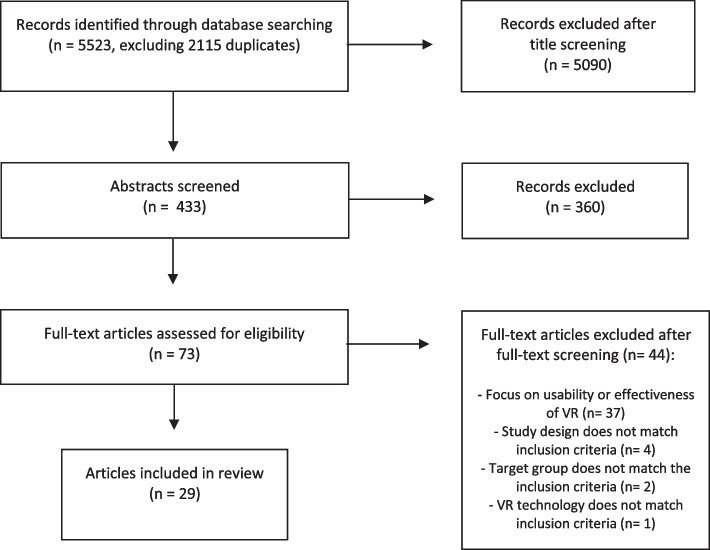
Search strategy and results

In the first step, two authors (MK & HK) screened all titles for assessment against the inclusion and exclusion criteria for the scoping review. Titles were included based on consensus between both authors. In the event of doubt or disagreement, the title was discussed by both authors. After screening the titles, both authors screened and assessed the abstracts using the inclusion and exclusion criteria. Abstracts were included or excluded based on consensus. In the final step, one author screened the full-text articles (MK). Reasons for excluding and any reservations about including were discussed with the other authors. The results of the search are reported in full and presented in a Preferred Reporting Items for Systematic Reviews and Meta-analyses (PRISMA) flow diagram [28] (Fig. 1).

### Data extraction strategy

The data extraction of this scoping review is mostly based on the guidelines of the Cochrane Handbook for Systematic Reviews of Interventions [29]. A systematic assessment of study quality was not performed because this review focused on giving a broad overview of all factors related to the implementation of VR. This resulted in a heterogeneous sample of included study topics and designs: ranging from explorative qualitative studies to reflective quantitative studies. The data extraction process started with the creation of a detailed data extraction form based on the research questions in Microsoft Excel. This form was generated to capture the most relevant information from all obtained studies and standardize the reporting of relevant information. The extracted data included the fields as presented in Table 1. One author (MK) filled out the data extraction forms; in case of uncertainties, a second author was consulted (HK). Secondly, for each category, relevant text fragments from each study were copied from the articles into the data extraction forms.

**Table 1:** Information extracted from included articles

**Category**	**Specification**
General information	General information regarding the authors, country, and year of publication
Study characteristics	Characteristics of the study; research question or goal of the study, study design, participants, and method of data collection
VR technology characteristics	Description of the VR technology and its goal, target group, and setting of use
Implementation characteristics	Description of theoretical implementation framework or model and implementation stage
Barriers (RQ1)	Barriers to implementation: factors that hinder the implementation of VR technology in healthcare settings
Facilitators (RQ2)	Facilitators to implementation: factors that help the implementation of VR technology in healthcare settings
Implementation objectives, strategies, and outcomes (RQ3 + 4)	Description of the implementation objectives, implementation strategies, and implementation outcomes
Recommendations (RQ5)	Recommendations or lessons learned to improve the implementation of VR technology in healthcare settings

### Data synthesis and presentation

To answer the first and second research questions, the fragments from the data extraction forms were coded inductively. To answer the third and fourth research questions, fragments were first coded deductively, based on the main categories of the NASSS framework: technology, adopters, organization(s), wider system or embedding, and adaptation over time [24]. Second, within these categories, the specific barriers and facilitators were coded inductively to identify recurrent themes. The implementation recommendations were coded inductively to answer the fifth and last research question. The first author executed the coding process, which included multiple iterations and constant adaptations until data saturation was reached. During this iterative process, multiple versions of the coding scheme were discussed with all authors and adapted accordingly.

## Results

### Search results

The search strategy, the number of included records, and the reasons for full-text exclusion are provided in Fig. 1. The main reason for excluding full-text articles was that studies focused on the usability or effectiveness of VR, rather than on the needs assessment, planning, execution, or lessons learned from the implementation process of VR.

### Study and technology characteristics

An overview of the characteristics of the 29 included records and the used VR technology is provided in Appendix 2. The following study designs were identified: qualitative (*n* = 13), quantitative cross-sectional (*n* = 10), and studies that used qualitative as well as quantitative methods (*n* = 6).

Of the 29 included records, 11 focused on VR use in rehabilitation clinics. Additional settings in which VR was applied are general health clinics, mental health clinics, or clinics for specific disorders, e.g., eating disorder clinics or burn clinics. The goal of VR technology was often to be of added value as a treatment tool. It was used to improve movement in rehabilitation patients (*n* = 11) or decrease anxiety in patients with a stress-related disorder (*n* = 2). In addition, it was applied to offer distraction or relaxation during medical procedures (*n* = 4). In addition to the variety in settings and applications of VR, the type of technology that was applied differed as well: from interactive VR (*n* = 26), in which patients can be immersed in a virtual environment, such as a shopping street or a restaurant, via a VR headset and interact with this environment, to (360°) videos (*n* = 4) in which patients are immersed in a virtual environment shown on a (computer) screen, with limited to no possibility for interaction.

### Implementation characteristics

An overview of the 29 included studies and the implementation characteristics, such as the use of an implementation model or the stage of implementation research are presented in Appendix 2. In this review, 8 of the 29 studies used a theoretical framework to structure implementation or data analysis. The Consolidated Framework for Implementation Research (CFIR) [30] was used in 3 studies and the Decomposed Theory of Planned Behavior (DTPB) [31] was also used in 3 studies. In addition, the Unified Theory of Acceptance and Use of Technology (UTAUT2) [32] was used in a single study, and the Innovation Diffusion Theory [33] was applied in one study as well.

Of the 29 included studies, the data collection of 12 studies took place before actual implementation and focused on factors, expected by stakeholders, that could influence future implementation. The data collection of the other 17 studies took place after actual implementation and reflected on existing factors related to implementation. Thus, most identified barriers, facilitators, and recommendations stated in this review were observed in studies that evaluated an existing implementation process.

### Barriers to implementation

Barriers to the implementation of VR were identified based on relevant fragments from the articles. In 26 records, a total of 69 different barriers were identified and divided into categories of the NASSS framework. All barriers are provided in Table 2. The barriers are explained in the accompanying text below.

**Table 2:** Barriers to implementation and the number of publications they were mentioned in (*n*)

**Category**	**Barrier**	**Definition**	***n***	**References**
**Category 1: Condition (** ***n*** ** = 13 barriers)**				
**Condition**	Cognitive limitations	A decline in cognitive capabilities, such as reasoning and problem-solving, could negatively affect VR use	6	[34–39]
	General decline	A decline in functional capabilities, such as mobility or communication, could negatively affect VR use	4	[35–38]
	Distress	VR use could induce distress and anxiety	4	[34, 38–40]
	Fatigue	Extreme fatigue in patients could negatively affect VR experience	1	[34]
	Dissociation	Experienced disconnection from themselves and the world could negatively affect VR experience	1	[34]
	Highly medicated	Effects of medication use could negatively affect VR use and experience	1	[39]
**Physical limitations**	Cybersickness	Motion- or cybersickness experienced while using VR	4	[13, 34, 39, 41]
	Issues with vision/hearing	Limited vision or hearing abilities could negatively affect VR use	3	[35, 36, 39]
	Epilepsy	VR use could trigger a seizure in patients with photosensitive epilepsy	2	[41, 42]
	Poor hand dexterity	Limited ability moving fingers and hands limits the use of VR controllers	1	[36]
	Wheelchair users	The use of a wheelchair can negatively influence movement in VR	1	[43]
**Socio-demographics**	Reluctance due to old age	Elderly can be less technology-aware and uncomfortable to use VR	7	[34, 37, 38, 44–47]
	Language barriers	Language can form a barrier when software is in another language	1	[37, 42]
**Category 2: Technology (** ***n*** ** = 11 barriers)**				
**Technical functionality**	Technical issues	Technical malfunctioning of VR hardware	9	[13, 40, 42, 43, 48–52]
	Lack of client safety	Lack of client safety due to unforeseen movement of patient in the treatment room while using VR	6	[13, 34, 44, 53–55]
	No reliable Wi-Fi	No reliable network connection which is necessary to use VR	4	[45, 46, 48, 50]
	Infection control issues	Difficult to control contaminations when using VR with multiple patients	3	[38, 39, 52]
	Data privacy and security	Lack of data privacy and security when using patient data in VR	2	[44, 51]
	System not charged	Battery of VR system is not charged and VR cannot be used	1	[50]
**Usability**	Lack of patient comfort	The use of VR headset and headphones could be uncomfortable	4	[34, 44, 52, 56]
	Usability issues	Issues with the usability and user-friendliness of VR	3	[34, 53, 55]
	Additional effort	The use of VR adds additional steps for healthcare providers during treatment	2	[13, 50]
**Effect of VR on treatment**	Isolation from contact	The VR headset can isolate patients from human contact	6	[40, 41, 45–47, 56]
	Lack of realism	Lack of realism and immersion experienced by patients in VR	4	[13, 40, 41, 57]
**Category 3: Value proposition (** ***n*** ** = 8 barriers)**				
**Influencing treatment**	Lack of personalization	Lack of personalization to treatment goals and patients’ needs	6	[13, 44, 50, 51, 58, 59]
	Distract from goals	The fun and gamification aspects of VR could distract from treatment goals	1	[48]
	Disinterest therapeutic activities	The preference for VR treatment could cause disinterest in other therapeutic activities	1	[48]
	No translation into real-world improvement	Treatment improvements in VR do not directly translate into real-world improvements outside of the treatment room	1	[51]
	Avoid in-vivo exposure	The use of VR can be a way to avoid in-vivo exposure	1	[40]
	Biased attention in group therapy	When VR is used in group therapy, the attention of the healthcare provider is focused on one patient and not on the other participants	1	[50]
**Practical resources**	Financial costs	Costs of purchasing and time for maintaining VR	9	[34, 38, 41, 42, 44–46, 51, 53]
	Time for maintaining	Lack of time for the maintenance and updating of VR software	3	[13, 42, 44]
**Category 4: Adopter system (** ***n*** ** = 18 barriers)**				
**Factors that influence**	Lack of research	Perceived lack of research and evidence on the added value of VR	10	[13, 34, 37–39, 41, 43, 44, 46, 58]
**Opinion towards VR**—healthcare providers	Lack of experience	Perceived lack of experience in working with VR	7	[35, 40, 43–46, 49]
	Lack of suitable patients	Perceived lack of appropriate patients that can be referred to VR treatment or perceived lack of support in referring patients	3	[13, 43, 60]
	Lack of support	Perceived lack of support from management in using VR	3	[43, 60, 61]
	Dissatisfaction with VR	Not satisfied with the use of VR hardware or software	2	[38, 49]
	No interest in VR use	Not interested in using VR technology in treatment	1	[58]
	Negative patient response	Expected negative patient response towards VR	1	[46]
	Resistance to new treatment	A general resistance towards new therapeutic approaches	1	[34]
**Factors that influence opinion towards VR**—patients	Low patient motivation	Patient motivation is low for VR treatment	5	[13, 35, 43, 51, 58]
	Stress inducing	The new aspects of VR technology could be stress inducing because patients are exposed to a new form of treatment and new reality	2	[35, 47]
	Disorientation during VR	Patient could experience disorientation when present in VR scenario	1	[52]
	Mistrust in new treatment	Patient mistrust in new or experimental treatment options	1	[44]
	No support healthcare provider	Patient does not feel supported in VR use by healthcare provider	1	[50]
**Integrating VR in routines**	Difficulty combining VR with existing treatment	Perceived difficulty combining VR with existing treatments and integrating VR in existing protocols	2	[36, 48]
**Knowledge and skills of healthcare providers**	Lack of time to learn and use VR	Lack of perceived time and opportunities to learn how to use VR and integrate VR in treatment	8	[13, 38, 42, 45, 46, 48, 50, 58]
	Lack of knowledge/skills	Lack of knowledge and skills to feel confident using VR	5	[34, 44, 46, 52, 53]
	Difficulty explaining VR	Difficulty explaining the VR system to patients during treatment	2	[37, 43]
	Difficult to learn VR	Difficult to learn how to use VR in treatment with patients	1	[40]
**Category 5: Organization (** ***n*** ** = 13 barriers)**				
**Readiness to innovate**	Other goals taking priority	Other goals that do not focus on VR taking priority within the organizational policy	1	[43]
	Negative culture towards innovation	Negative organizational culture towards innovation and new technology	1	[44]
**Introducing VR to healthcare providers**	No opportunity to try VR	Not giving an opportunity to healthcare providers to try out VR for themselves	8	[13, 34, 37, 38, 47, 48, 50, 54]
	Lack of education	Not organizing enough educational opportunities to learn how to use VR	2	[13, 37]
	Lack of training courses	Not offering enough standardized training courses to healthcare providers	2	[38, 41]
**Providing support for healthcare providers**	Lack of time to learn VR	Not making enough time available for healthcare providers to learn how to use VR	13	[13, 37, 38, 42–46, 48, 50, 53, 58, 59]
	Lack of technical support	Not offering technical support to help set up the VR system or help fix hardware or software malfunctions	12	[13, 34, 38, 42–46, 50, 53, 58, 60]
	Lack of rooms	Not having enough rooms available for VR treatment	8	[13, 35, 37, 38, 44, 48, 50, 58]
	Insufficient VR systems	Not having enough VR systems available for VR treatment	3	[13, 35, 38]
	No official channels to report performance issues	Not creating official channels to report performance issues experienced during VR treatment	1	[50]
**Integrating VR in workflow**	Lack of guidelines on patient suitability	Lack of guidelines on suitability of patients and medical indication for VR treatment	3	[34, 44, 50]
**Providing conditions for use**	Lack of treatment protocols	Lack of validated treatment protocols on how to use VR in treatment	2	[41, 42]
	Integration of VR	Not integrating VR in existing workflows and traditional treatment	1	[44]
**Category 6: Wider system (** ***n*** ** = 3 barriers)**				
**Societal development**	Not innovation minded	Opinion leaders are not innovation-minded and do not support VR	2	[44, 46]
	Focus on well-being over treatment for specific conditions	Health industry’s focus on creating VR for general wellbeing over developing VR treatments for specific conditions	1	[44]
**Regulatory/legal issues**	Ethical or legal concerns	Ethical or legal concerns around the use of VR in treatment, such as cybersecurity, privacy and regulations	1	[51]
**Category 7: Embedding and adoption over time (** ***n*** ** = 3 barriers)**				
**Challenge to scale up**	Lack of insurance reimbursement	Lack of insurance reimbursement to compensate costs of VR use	2	[44, 53]
	Sustainability	VR use is not sustainable over a longer period of time, because risk of hardware quickly becoming obsolete	2	[41, 59]
	Lack of technical support	Lack of technical support to maintain hardware limits upscale of VR use	1	[42]

A broad range of barriers was relevant to the implementation of VR in healthcare. Most identified barriers were related to the organization category of the NASSS framework. These were mainly focused on the lack of practical resources for healthcare providers to use VR. For example, the organization does not schedule sufficient time for healthcare providers to learn how to use VR and how to integrate VR into practice. In addition to a lack of time, not enough technical support, treatment rooms for VR, and VR equipment to treat patients were mentioned as organizational barriers.

Frequently mentioned barriers related to the adopters were factors that negatively influence healthcare providers’ opinions of VR. First, a lack of research and evidence on the added value of VR was mentioned as a barrier. Second, a perceived lack of experience in working with VR was said to cause a lack of confidence and self-efficacy in healthcare providers to work with VR during treatment. The perceived lack of time and limited opportunities to learn how to use VR contributed to this feeling.

Furthermore, technical barriers were identified to hinder VR implementation. Functional issues, such as technical malfunctioning of VR hardware or software, or a lack of client safety while wearing a VR headset in the limited space of the treatment room that limits freedom of movement were most frequently mentioned as barriers. Related to the VR headset, a lack of physical comfort for the patient when wearing the VR headset and the feeling of isolation while wearing the headset were frequently mentioned as barriers.

Lastly, barriers related to the condition, value proposition, wider system, and embedding and adoption over time categories of the NASSS framework were less frequently identified. The conditions and physical limitations of patients that could negatively influence VR use, such as several cognitive limitations, distress, or cybersickness during VR, were mentioned as barriers. Related to the value proposition, barriers such as high costs to purchase VR equipment or the lack of time for maintaining the VR hardware were mentioned. In addition, the lack of personalization to patients’ needs and treatment goals was mentioned as a barrier. The barriers related to the wider system and adoption over time, such as organizations not being innovation-minded or the lack of insurance reimbursement to compensate for costs of VR use, were mentioned less frequently.

### Facilitators to implementation

Besides barriers, a total of 53 different facilitators to the implementation of VR in healthcare were identified in 26 records. Facilitators were identified based on relevant fragments from the articles and are divided into categories of the NASSS framework. They are mentioned and explained in Table 3 and the accompanying text below.

**Table 3:** Facilitators to implementation and the number of publications they were mentioned in (*n*)

**Category**	**Facilitator**	**Definition**	***n***	**References**
**Category 1: Condition (** ***n*** ** = 1 facilitator)**				
**Socio-demographics**	Young age	Younger people may be more open to new technology and feel comfortable to use VR during treatment	2	[34, 38]
**Category 2: Technology (** ***n*** ** = 5 facilitators)**				
**Technical functionality**	Client safety	Client is physically safe in treatment room while using VR hardware	6	[13, 34, 44, 53–55]
	Reliability	VR hardware is reliable and stable while in use	1	[55]
**Usability**	Patient comfort	The patient is comfortable while using VR hardware and software	4	[34, 44, 52, 56]
	Easy to use	The VR hardware and software is easy to use by end-users	3	[34, 53, 55]
**Effect of VR on treatment**	Realism and immersion	VR is able to induce feelings of realism and immersion	4	[13, 40, 41, 57]
**Category 3: Value proposition (** ***n*** ** = 8 facilitators)**				
**Influencing treatment**	Safe and controlled environment	Having a virtual environment that is controlled by the healthcare provider and therefore offers a safe space to practice behavior	5	[39–41, 45, 51]
	Different reality	Practice behavior in a virtual environment of choice, while physically in the treatment room	4	[41, 44, 48, 51]
	Personalizing treatment	VR can be adapted to fit patient needs and treatment goals	3	[51, 54, 55]
	Facilitate human interaction	VR could facilitate human contact by practicing virtual roleplays, which other technologies can not	3	[39, 45, 47]
	Control and self-efficacy	VR could increase behavioral control and self-efficacy in patients	2	[41, 51]
	Insight into behavior and experiences	VR could increase insight of healthcare provider into behavior of patients and their experiences	1	[34]
**Practical resources**	Financial viability	VR demonstrates financial viability and has a strong business case	1	[34]
	Time and resource efficient	VR is time and resource efficient to use compared to other treatment forms	1	[40]
**Category 4: Adopter system (** ***n*** ** = 18 facilitators)**				
**Factors that influence opinion towards VR**—healthcare providers	Evidence of VR value	Availability of validated evidence on the value of VR for treatment	10	[13, 34, 37–39, 41, 43, 44, 46, 58]
	Experience with technology	Having experience with technology in general and/or with VR	7	[35, 40, 43–46, 49]
	Added value of VR	Being aware of the benefits of VR for patients and treatment	4	[34, 36, 46, 48]
	Improvement in patients	Perception of improvement in patients’ health and treatment goals	2	[37, 50]
	Satisfaction with VR	Being satisfied with the usability of VR hardware and software	2	[38, 49]
	Support from management	Perceived support from management to use VR	2	[43, 58]
	Innovativeness	Being intrigued by the innovativeness of VR in existing treatment	1	[45]
**Attitude towards VR**—patients	Patient motivation	VR could enhance patient motivation and engagement in treatment	11	[13, 34, 35, 40, 43, 48, 50, 51, 53, 57–59]
	Positive	VR is perceived as positive, fun and engaging by patients	1	[50]
	Less stressful	Exposure in VR is less stressful than in-vivo exposure	1	[51]
	Encouraged by healthcare provider	Patient feels encouraged and supported by healthcare provider to use VR	1	[50]
**Attitude towards VR**—colleagues	Positive social influence	Positive opinion of VR of colleagues causes a “domino effect”	1	[37]
**Integrating VR in routines**	Combine and integrate VR	The capacity to combine and integrate VR in existing treatment	2	[36, 48]
**Knowledge and skills needed to use VR**	Training	Offering training on how to use VR hardware and software	8	[34, 35, 37, 39, 45, 46, 48, 50]
	Knowledge and skills	Developing sufficient knowledge and skills to feel confident and comfortable using VR	5	[34, 44, 46, 52, 53]
	Intervision	The possibility of frequent contact with colleagues on VR for support, troubleshooting and reviewing VR use	2	[13, 45]
	Protocols	Having protocols or guides available on how to use VR	2	[46, 60]
	Technological capabilities	The use of VR may increase technological capabilities and vice versa	1	[45]
**Category 5: Organization (** ***n*** ** = 18 facilitators)**				
**Readiness to innovate**	Innovative culture	Having an innovative culture within the organization	2	[34, 44]
	Champions	Strategic recruitment of champions to promote VR uptake and credibility	2	[34, 44]
	Willingness to invest	Organization is willing to invest time and money in VR	1	[58]
**Introducing VR to healthcare providers**	Try out VR	Giving the opportunity and time to healthcare providers to try out VR for themselves	8	[13, 34, 37, 38, 47, 48, 50, 54]
	Educational materials	Creating access for healthcare providers to educational materials on VR	5	[43, 46, 54, 58, 60]
	E-mail updates	Sending e-mail updates on VR use and added value of VR to keep healthcare providers informed on VR progress in the organization	2	[13, 60]
	Staff meetings	Introduce VR and opportunities of VR during staff meetings	1	[34]
**Providing support for healthcare providers**	Time to learn VR	Offering enough time for healthcare providers to learn how to use VR	13	[13, 34, 38, 42–46, 50, 53, 58, 60]
	Technical system support	Offering technical system support to healthcare providers who work with VR	12	[13, 34, 38, 42–46, 50, 53, 58, 60]
	Rooms availability	Having enough rooms available for VR use	8	[13, 35, 37, 38, 44, 48, 50, 58]
	Support staff	Having support staff available that helps set up the VR system	6	[35–37, 43, 45, 58]
	VR systems	Sufficient VR systems to use in treatment	5	[13, 35, 38, 43, 56]
	Staff who operate VR	Having selected healthcare providers available who operate VR for multiple patients, instead of training all staff on VR	4	[35–37, 58]
	Staff who supervise VR	Having technical support staff available who supervise VR sessions and help healthcare providers	4	[37, 38, 45, 53]
	Training on patient suitability	Organizing training in determining patient suitability for VR	4	[34, 35, 39, 59]
	Train-the-trainer	A learning model in which colleagues who have experience with VR train colleagues that are new to VR	1	[59]
**Integrating VR in organizational structure and workflow**	Fit current protocols	VR should fit with current treatment protocols	2	[42, 44]
	Reinforcement from management	Reinforcement from management to refer clients to VR treatment	2	[35, 37]
**Category 6: Wider system (** ***n*** ** = 2 facilitators)**				
**Societal development**	Innovation minded	Opinion leaders being innovation-minded and open towards VR	2	[44, 46]
	Opinion of society	General positive opinion of society on VR	1	[41, 46]
**Category 7: Embedding and adoption over time (** ***n*** ** = 1 facilitator)**				
**Challenge to scale up**	Commonplace and affordable	VR becomes more commonplace and affordable, making it easier to scale up VR use	1	[41]

In comparison to the barriers, facilitators to implementation were identified less frequently in the included studies. Similar to the barriers, most facilitators were related to the organization category of the NASSS framework. As an organization, providing support, time, room, and technical system support to healthcare providers to learn and use VR were mentioned most frequently as facilitators.

In multiple studies, it was mentioned that adopters of VR technology need training and education on how to use and integrate VR into treatment. Healthcare providers want to increase their knowledge, skills, and experience with VR to feel confident and increase self-efficacy in using VR in treatment with patients. Besides, as a facilitator in the adopter’s category, it is mentioned that having access to evidence on the added value of VR for treatment is a major facilitator in VR implementation because healthcare providers feel the use of VR is validated within the treatment.

Lastly, facilitators in the condition, technology, value proposition, wider system, and embedding and adoption over time category of the NASSS framework were identified less frequently. For example, when looking at the sociodemographic factors of patients, the young age of patients was identified as a facilitator since these people tend to be more open to new technology and treatments and feel more comfortable using VR. Related to technology, ensuring client safety was mentioned as a facilitator, that is creating a physically safe space in the treatment room for patients to use VR. This safe and controlled environment was also identified in the value proposition category. Meaning that healthcare providers can create a safe space for patients to practice challenging behavior. Lastly, being innovation-minded as an organization and VR becoming more and more commonplace and affordable to scale up were both mentioned as facilitators in the wider system category and the adoption over time category of the NASSS framework.

### Implementation strategies, objectives, and outcomes

An overview was created of the implementation strategies, objectives, and outcomes that were extracted from the included studies (see Appendix 2). In two studies, a clear implementation objective was mentioned [13, 43]. These objectives both focused on designing an implementation intervention, the knowledge translation (KT) intervention, to translate knowledge about the use of VR to the healthcare provider. In addition, they aimed to identify factors that influenced VR adoption and healthcare providers’ support needs.

Of the 29 included records, 8 studies described actual implementation strategies [13, 34, 35, 43, 44, 48, 53, 60]. Most were mentioned in studies that collected data after implementation and reflect on existing implementation processes. In the included studies that described expected implementation factors, implementation strategies were most often not described. These studies focused on identifying potential barriers and/or facilitators in preparation for the implementation phase and did not evaluate the used strategies.

A summary of the described implementation strategies mentioned in the included records is displayed below in Table 4. Examples of strategies focused on practical resources were VR equipment to be used in treatment, treatment rooms in which the VR technology can be set up and used, and time for healthcare providers to learn about VR use. In addition, training and education on VR use were mentioned as important strategies. Hands-on interactive training, e-learning modules, mentorship for support and troubleshooting, and matching protocols and guidelines on how to use VR were mentioned. To set up VR treatment, an identified implementation strategy is to give support to healthcare providers in selecting appropriate content in VR that fits the patient’s needs and give information on how to instruct the patient about VR treatment. Lastly, implementation strategies that help to increase the motivation of healthcare providers to use VR were addressed. For example, having sufficient time to discuss the potential and added value of VR or having support from champions or mentors, experienced healthcare providers who share their experience with VR, to motivate others to integrate VR into their treatment practice were used during implementation.

**Table 4:** Summary of implementation strategies mentioned in included records

**Category**	**Implementation strategies**	**References**
Practical resources	• Purchasing VR equipment	[35]
	• Availability of treatment rooms	[13]
	• Availability of treatment manuals and protocols on how to use VR in practice	[13, 34]
	• Availability of time to reflect and understand how to use VR	
	• Availability of time to set up and maintain the VR system	[35]
	• Hiring support staff to use VR, to coach clinicians in the use of VR, or to maintain the VR system	[44]
Education on VR use	• Training (in person and online) to learn how to use VR and discussing appropriate content in treatment	[43, 53, 58]
	• E-learning modules on foundational knowledge about clinical VR use and the added value of VR	[58]
	• Hands-on learning (discuss VR experience, training sessions, and case scenarios)	[58]
	• Experiential learning (discuss and reflect on VR use)	[58]
Setting up VR treatment	• Having an overview of the content (specific environments, exercises, games) that is available in VR	[43]
	• Having information on how to select goal-appropriate content for clients and their treatment goals	[43]
	• Getting familiar with the VR technology to instruct patients in its use	[13]
Increasing motivation to use VR	• Availability of mentors/champions to contact with questions or support needs and clinicians with VR experience who can share both evidence for the added value of VR and successful treatment experiences	[44, 58]
	• Didactic reminders (weekly e-mails with tips for VR use)	[58]
	• Self-directed research	[53]
	• Spending time considering the added value of VR in practice	[13]

The explicit conceptualization of implementation outcomes and the use of these outcomes to formulate implementation objectives or design implementation strategies was not described as such in the included records. The concepts of acceptability, adoption, uptake, or feasibility were mentioned in 12 records (see Appendix 2); however, they were not integrated as outcomes into a systematic implementation process.

### Recommendations for implementation

In Table 5, an overview of the 51 different recommendations for the implementation of VR in healthcare that were mentioned in 20 records is provided. These recommendations were inductively coded and divided into seven categories: (1) Increase understanding of patient suitability, (2) Improve knowledge and skills on VR use, (3) Improve healthcare providers’ engagement with VR, (4) Have support staff available, (5) Points of attention for developing VR treatment, (6) Support functionality of VR hardware and software, and (7) Design and development of implementation.

**Table 5:** Recommendations on implementation and the number of publications they were mentioned in (*n*)

**Category**	**Code**	**Specification**	***n***	**References**
**Category 1: Increase understanding of patient suitability (** ***n*** ** = 3 recommendations)**				
**Understanding patient suitability**	Understanding suitability	Determining for which patients VR treatment is fitting	6	[34, 38, 43, 52, 54, 58]
	Functional limitations	Take patients’ functional limitations into account, such as mobility or communication skills, before referring patients to VR	2	[43, 54]
	Not mandatory	Consider that not all patients want to use VR; it should not be mandatory to use	1	[62]
**Category 2: Improve knowledge and skills on VR use (** ***n*** ** = 17 recommendations)**				
**Learning how to use VR**	Training programs	Offer training programs on technical skills for healthcare providers	7	[34, 35, 38, 39, 45, 48, 51]
	Educational resources	Develop and disseminate quality educational and training activities and materials	5	[34, 39, 45, 48, 60]
	Mentorship	Offer mentorship by colleagues experienced in VR use	4	[38, 48, 54, 59]
	Multi-phased	Develop multi-phased strategies to address healthcare providers needs as they progress from novice to experienced VR users	3	[38, 43, 60]
	Decision-making	Develop training on clinical decision-making and application competences of when to use VR and for whom	3	[38, 43, 48]
	Frequently reassess	Frequently reassess multi-phased strategies to see if the strategies fit with the needs of healthcare providers and patients	3	[35, 43, 48]
	Different formats	Use different formats in training (e.g., written documentation, video, online activities)	2	[13, 38]
	Online vs. real-life	Combine online and real-life training on VR use for healthcare providers	2	[13, 38]
	Individual vs. group	Combine individual and group learning on VR use for healthcare providers	2	[13, 38]
	Train-the-trainer	Use the train-the-trainer model in which colleagues who have experience with VR train colleagues that are new to VR	2	[36, 43]
	Comfortable	Make healthcare providers comfortable with VR use by letting them try out VR and experiment with colleagues	2	[50, 60]
	Refresher sessions	Include refresher sessions between initial skills training and healthcare providers first use of VR with patients	1	[13]
**Information provision on VR**	Knowledge gaps	Address healthcare providers’ knowledge gaps and misconceptions about VR and address the added value of VR	2	[34, 48]
	Acceptability	Address acceptability and feasibility to aid adoption and sustained uptake	1	[34]
	Theoretical background	Provide theoretical background on VR use and effect on treatment outcomes	1	[39]
	Protocols	Develop guidelines and treatment protocols	1	[34]
**Available time**	Support time	Management should support time for training, use and maintenance of VR	1	[48]
**Category 3: Improve healthcare providers’ engagement with VR (** ***n*** ** = 4 recommendations)**				
**Awareness and information on added value of VR**	Benefits	Communicate possible benefits and the importance of VR and its possible contributions to treatment to healthcare providers and patients	4	[36, 49–51]
	Evidence	Use example cases and supporting evidence of added value of VR from research	2	[36, 52]
	Experience	Let healthcare providers experience VR to see the potential and increase motivation for use	1	[36]
	Purpose	Inform about purpose of using VR	1	[45]
**Category 4: Have support staff available (** ***n*** ** = 3 recommendations)**				
**Support staff**	Staff support	Hire staff to support VR use and maintenance	4	[36, 44, 49, 52]
	Champions	Use other experienced healthcare providers or mentors to promote uptake and increase self-efficacy	3	[34, 38, 59]
**Motivation**	Encouragement	Organization should provide encouragement to healthcare providers with regard to using VR and motivate them to expanding their skills	1	[49]
**Category 5: Points of attention for developing VR treatment (** ***n*** ** = 11 recommendations)**				
**Treatment considerations**	Frequency of use	Use of VR in treatment ranging from daily to once a week	2	[42, 54]
	When to use	Introduce VR early in treatment, but not at the first appointment, because the use of VR can be overwhelming	2	[42, 62]
	Establish goals	Establish measurable goals for VR treatment	1	[43]
	Match patient needs	VR treatment should match patient needs	1	[58]
	Become familiar	Patients should spend sufficient time with VR technology before treatment starts to become familiar with the system	1	[39]
	Step by step	Start step by step and slowly navigate within the virtual environment	1	[39]
**Safety**	Freedom of movement	Treatment room should offer sufficient freedom of movement to keep risk of falling as low as possible	1	[54]
	Switch off	VR systems should be able to switch off immediately, e.g., in case of dizziness	1	[54]
	Infection control	Consider hygienic measures before implementing VR in practice	1	[52]
**Integration into workflow**	Part of treatment	Offer VR as part of existing treatment	2	[48, 54]
	Knowledge Translation intervention	Support clinical integration of VR by knowledge translation intervention	1	[38]
**Category 6: Support functionality of VR hardware and software (** ***n*** ** = 9 recommendations)**				
**Functionality**	Clarify needs	Clarify functional needs of VR technology that are necessary in use according to healthcare providers	1	[36]
	Works as intended	Check if technology works as intended	1	[36]
**Technical issues**	Channels to report	Make sure that healthcare providers are aware of the official channels that they can use to report technical issues	1	[50]
**Software**	Patient-appropriate	Create patient-appropriate content for VR software that fits patient needs	3	[39, 43, 52]
	Setting-appropriate	Create setting-appropriate content for VR software that fits the setting	2	[52, 62]
	Age-appropriate	Create age-appropriate content that fits patient age	1	[62]
**Hardware**	Interaction	Interaction between healthcare provider and patient should still be possible with headset on	1	[62]
	Relocatable	System has to be practical to set up in a treatment room and easy to relocate if necessary	1	[50]
	Adaptable	System has to be able to adapt for limited mobility of patients	1	[62]
**Category 7: Design and development of implementation (** ***n*** ** = 4 recommendations)**				
**Using a theoretical framework**	Guide development	Use a theoretical framework to guide development of relevant implementation strategies to enhance uptake	1	[34]
**Implementation intervention**	Intervention	Use a multi-model and active implementation intervention to support needs of stakeholders and address barriers to VR use	2	[38]
**Engaging stakeholders**	Key stakeholders	Engage key stakeholders during the design and development process of implementation	4	[34, 36, 50, 59]
**Integration of VR in workflow**	Understanding needs	Understand clinical reasoning processes and treatment needs as means of informing features and functionality of VR systems that support integration in practice	2	[38, 59]

The first recommendation was to increase the understanding of patient suitability. In other words, it should be clear for healthcare providers how they can determine for which patients VR treatment is a fitting option. One way to determine patient suitability is to take into account the functional limitations of patients, such as their level of mobility or communication skills, before referring patients to VR treatment. Next to functional limitations, one should take into account cognitive limitations and any sensitivity to cybersickness. Patient suitability can be dependent on the goal of VR treatment, as some functional or cognitive limitations are not always a barrier to VR use.

The second recommendation was to improve the knowledge and skills of healthcare providers on VR use. Training programs and other educational resources, such as training days, online meetings, or instruction videos, that should be developed and disseminated to healthcare providers were mentioned as key elements to improving knowledge and skills.

The third recommendation was to improve healthcare providers’ engagement with VR. To accomplish this, the benefits of VR use and its possible contributions to treatment should be communicated to healthcare providers and patients. The use of successful example cases and disseminating supportive evidence of the added value of VR were mentioned as options to increase the engagement of healthcare providers with VR.

The fourth recommendation was to have sufficient support staff available to support VR use during treatment and maintain VR equipment. In addition, champions or mentors, healthcare providers experienced in VR treatment, were mentioned to promote uptake and increase the self-efficacy of other healthcare providers in VR use.

The fifth recommendation was related to developing VR treatment. The included studies gave some inconsistent suggestions on the frequency of use, from daily to once a week. Important aspects of developing a VR treatment are to set clear treatment goals, let the patient become familiar and comfortable with the VR equipment and software, and increase the treatment difficulty step by step.

The sixth recommendation was to support the functionality of VR hardware and software and ensure that it fits the user. Software should be appropriate for the patient’s needs, and age, and should fit the treatment setting. For example, VR software for forensic mental healthcare patients with aggression regulation problems should be able to let patients practice self-regulation strategies in virtual environments in which their undesired behavior is triggered. This could be a bar or supermarket with strangers for one patient, or a more intimate setting with a partner at home for another. The hardware needs to be adaptable for the limited mobility of patients, for example, patients that are wheelchair-bound. In addition, the VR hardware should still give the possibility for healthcare providers and patients to interact during the use of VR. The patient needs to be able to hear the voice of the healthcare provider.

The seventh and last recommendation was related to the design and development of the implementation of VR in practice. In multiple studies, it was advised that healthcare organizations use a structured, multi-model implementation intervention to support the needs of stakeholders and address barriers to VR use. The key stakeholders should be engaged during the development process of implementation interventions. It was recommended to use a theoretical framework, such as the Consolidated Framework for Implementation Research (CFIR) [46] or the Decomposed Theory of Planned Behavior (DTPB) [47] to guide the development of relevant implementation strategies to enhance the uptake of VR in healthcare practice.

## Discussion

### Principal findings

This scoping review was conducted to provide insight into the current state of affairs regarding the implementation process of virtual reality in healthcare and to identify recommendations to improve implementation research and practice in this area. This review has resulted in an overview of current implementation practices. A broad range of study designs was identified: from qualitative studies that described expected factors of implementation, to quantitative methods that summarized observed factors. From the included studies, it can be concluded that the main focus of the implementation of VR is on practical barriers and facilitators, and less attention is paid to creating a systematic implementation plan, including concrete implementation objectives, developing suitable implementation strategies to overcome these barriers, and linking these barriers or facilitators to clear implementation outcomes. Only two studies described objectives for implementation and the practical strategies that were used to reach these objectives. Most implementation strategies that were described were related to practical resources and organizational support to create time and room for healthcare providers to learn about VR and use it in treatment. Despite differences in the type of VR technology, healthcare settings, and study designs, many studies identified the same type of barriers and facilitators. Most identified barriers and facilitators focused on the adopter system and organization categories of the NASSS framework [24], e.g., the needs of healthcare providers related to VR use and the organizational support during the implementation of VR. The most frequently mentioned barriers were a lack of practical resources, a lack of validated evidence on the added value of VR, and a perceived lack of experience in working with VR. This review showed that facilitators were studied less than barriers. Most of the included studies only described the implementation barriers. However, in the studies that did mention facilitators, similar themes were found between identified barriers and facilitators, mostly related to practical resources, organizational support, and providing evidence of the added value of VR were found. The content of the recommendations for the implementation of VR fits with the foregoing.

### Comparison with prior work

Despite the importance of concrete strategies to successfully implement VR [20] and the conceptualization of implementation outcomes to understand the process and impact of implementation [22], there is a lack of research on this systematic implementation approach. In this review, only a few studies used a theoretical framework to structure implementation or data analysis. Frameworks that were mentioned most often were the Consolidated Framework for Implementation Research (CFIR) [30], and the Decomposed Theory of Planned Behavior (DTPB) [31]. However, none of the studies that mentioned the use of these models described an explicit link between the separate strategies, barriers, or facilitators and the integrated systematic implementation process. This illustrates the gap in research between identifying factors that influence implementation and linking them to practical strategies and implementation outcomes to form a coherent implementation intervention. The development of a coherent implementation intervention was only mentioned in two studies that were included in this review. To illustrate, one study set up an implementation intervention that promotes clinician behavior change to support implementation and improves patient care [63]. A coherent intervention could be an option to structure the implementation process and bridge the gap between knowledge of the use of VR to actual uptake in practice [63]. However, from implementation frameworks, such as the NASSS framework [24] or the CFIR [30], it is clear that the focus should lie on a coherent multilevel implementation intervention that focuses on all involved stakeholders and end-users, not only on one stakeholder.

The importance of focusing on the behavior change of all involved stakeholders, such as healthcare providers, patients, support staff, and managers, is reflected in the results of this review. Most barriers, facilitators, strategies, and recommendations are related to stakeholders within the healthcare organization that need to change their behavior in order to support implementation. For example, healthcare providers are expected to learn new skills to use VR and organizational management needs to make time and room available to support healthcare providers in their new learning needs and actual VR use during treatment. This highlights the importance of focusing on strategies that target concrete behavior of stakeholders for successful implementation. Identifying concrete behavior that is targeted in an implementation intervention can help describe who needs to do what differently, identify modifiable barriers and facilitators, develop specific strategies, and ultimately provide an indicator of what to measure to evaluate an intervention’s effect on behavior change [64]. The focus on behavior in implementation is not new, it is an important point of attention in the implementation of other eHealth technology [14]. However, based on the results of this scoping review, this focus is lacking in research on VR implementation.

To design implementation interventions that focus on the behavior change of stakeholders, existing intervention development frameworks can be used. An example is Intervention Mapping (IM). Intervention Mapping is a protocol that guides the design of multi-level health promotion interventions and implementation strategies [65, 66]. It uses a participatory development process to create an implementation intervention that fits with the implementation needs of all involved stakeholders [65]. Eldredge et al. [65] and Donaldson et al. [67] IM can provide guidance on overcoming barriers by applying implementation strategies based on behavioral determinants and suitable behavior change techniques [65]. For example, when reflecting on the implementation strategies described in this review, providing feedback as a behavior change method can be used during the education or training on VR use to support the learning needs of healthcare providers. In addition, providing opportunities for social support could be seen as the behavior change technique behind the need for support and discussion of VR use during intervision groups with other healthcare providers.

### Implications for practice and future research

The results from this review provide various points of departure for future implementation research and implications for practice. An important implication for both is the need for a systematic approach to the implementation process. Most studies identified in this review focused only on barriers or facilitators to implementation, not paying attention to the systematic process of developing an implementation intervention that specifies implementation objectives, describes suitable strategies that fit with these barriers and facilitators, and conceptualizes implementation outcomes to evaluate the effectiveness of these strategies. The development of an implementation intervention should preferably be supported by theoretical implementation frameworks such as the Consolidated Framework of Implementation Research [30], or the NASSS framework [24]. In this review, all implementation factors could be coded with and analyzed within the categories of the NASSS framework. Indicating its usefulness in structuring implementation research. Future research could focus on applying and evaluating such implementation frameworks to the implementation of VR in healthcare, specifying factors related to the implementation of VR and focusing on all phases and levels of implementation.

In addition, it could be valuable to focus on existing intervention development frameworks, such as Intervention Mapping, to guide the design of a complete implementation intervention. Future research could apply these existing frameworks in an implementation context, reflect on the similarity in working mechanisms and evaluate their influence on the implementation process and the behavior change of the involved stakeholders. This way, a first step in identifying the added value of systematic implementation intervention development can be made.

Furthermore, as being aware and convinced of the added value of VR within the treatment of patients is seen as an important facilitator of implementation for healthcare providers and organizations, it would be valuable for future research to focus on the evaluation of the efficacy of VR within healthcare practice. However, this raises an interesting paradox. Healthcare organizations and healthcare providers would like to have evidence of the added value of VR before investing in the technology for its implementation, but the efficacy of VR in practice can only be determined in an ecologically valid way when it is already thoroughly implemented in healthcare practice.

### Strengths and limitations

This review set out to give an overview of factors that are related to the implementation practice of VR in healthcare. A strength of this study is that it used the NASSS framework to structure the analysis and review process. The use of an implementation framework contributed to systematic data collection and analysis, which can increase the credibility of the findings [68]. However, the use of the NASSS framework also revealed some drawbacks. Although all implementation factors were categorized within the categories of the NASSS framework, this coding was limited by the description of these categories and the overlap between some categories. For example, most barriers and facilitators that were categorized under organization, adopters, or technology were relevant for sustainable embedding and thus could fit in the category “embedding and adaptation over time” as well. In addition, the description of the category “condition,” the illness of the patient, and possible comorbidities, which are often influenced by biomedical and epidemiological factors [24], is too limited to describe all factors related to patient suitability for VR. The condition of a patient within mental healthcare is often related to other aspects, such as sociodemographic factors like age, technical skills, and feeling comfortable using new technology. All these factors could influence patient suitability for VR. Besides, in most included studies, the barriers or facilitators were not described in great detail, which made the coding process within the NASSS categories more difficult.

Furthermore, when titles of screened records did not focus on the implementation process of VR, e.g., studies that only focused on usability or effectiveness, they were excluded. Since usability studies could still partly focus on implementation, this may have caused us to miss publications that could provide interesting insights on implementation but whose main focus was other than that. We tried to overcome this limitation by selecting detailed inclusion and exclusion criteria for the literature search and abstract screening. The study was excluded only when there was no indication of a link between usability and implementation.

In addition, the full-text screening and data-extraction process were executed by one researcher. This could have caused us to miss information related to the topic. However, since the researcher used inclusion criteria that were thoroughly discussed during the title and abstract screening, and used a detailed data-extraction form, the chances of missing information are considered to be low. Furthermore, the first and second authors both extracted data from a few full-text articles, and in case of doubt, full-text were discussed with both authors.

Furthermore, because this scoping review aimed to provide an overview of the current state of affairs related to the implementation of VR in healthcare, all available studies were included, regardless of their quality and type of results. This is in line with the general aim of scoping reviews, which is to present a broad overview of the evidence on a topic. Since a quality assessment was not conducted, not all results of included studies might be valid or reliable. In addition, most of the barriers, facilitators, and recommendations stated in this review are observed in studies that took place after actual implementation. However, some of these factors were mentioned as potential factors related to implementation in studies that collected data before actual implementation. These factors were described as expected factors by involved stakeholders, but not observed. Therefore, these findings should be interpreted with care.

### Conclusion

This scoping review has resulted in an initial overview of the current state of affairs regarding the implementation of VR in healthcare. It can be concluded that in the included publications, a clear focus on practical barriers and facilitators to the implementation of VR has been identified. In only a few studies implementation frameworks, specified strategies, objectives, or outcomes were addressed. To take the implementation of VR in healthcare to the next level, it is important to ensure that implementation is not studied in separate studies focusing on one element, e.g., therapist-related barriers, but that it entails the entire process, from identifying barriers to developing and employing a coherent, multi-level implementation intervention with suitable strategies, clear implementation objectives and predefined outcomes. This implementation process should be supported by implementation frameworks and ideally focus on behavior change of stakeholders such as healthcare providers, patients, and managers. This in turn might result in increased uptake and use of VR technologies that are of added value for healthcare practice.

## Data Availability

All dataset(s) supporting the conclusions of this article are available in the included primary studies.

## Appendix 1. Full electronic search strategy

### Search terms

**Table:**

Set	Key concepts	Related terms
*Set 1*	***Implementation***	**Adoption, dissemination, introduction, uptake**
*Set 2*	***Virtual reality (VR)***	**VR, Virtual technology, virtual environment**
*Set 3*	***Health care***	**Health, care, treatment**

### Search string

TS = (implement* OR adopt* OR disseminat* OR introduc* OR “uptake”) AND TS = (“virtual reality” OR VR OR “virtual technolog*” OR “virtual environment”) AND TS = (health* OR “care” OR treat*)

## Appendix 2. Study, technology, and implementation characteristics per study

**Table:** Table 6 Study characteristics, characteristics of VR technology, and implementation characteristics per study

Authors, year, country	Study	VR technology	Implementation
	Study goal, design, and participants	VR technology, goal, target group, and setting	Implementation stage, strategies, target group, objectives, and outcomes
Algahtani, Altameem, and Baig, 2021; Saudi Arabia [49]	**Goal**: The study explores the current state of VR technology adoption, factors that influence such adoption, and the extent of this technology’s efficiency when it is used for vaccinating children **Design**: Quantitative cross sectional, experimental **Participants**: Workers in vaccination clinics (*n* = 186) survey—and pediatric patients (*n* = 6) experiment **Data collection**: Survey and experiment	**VR technology and goal:** VR eyewear that shows an amusing video that distracts children during vaccination **Target group VR:** pediatric patients **Setting:** Vaccination clinic	**Stage**: After implementation **Target group implementation**: Workers in health centers **Objective**: - **Model**: UTAUT2 **Strategies**: N/S **Outcomes**: Adoption, satisfaction, behavioral intention
Banerjee-Guénette, Bigford, and Glegg, 2020; Canada [60]	**Goal**: Develop and evaluate the impact of a multifaceted KT intervention (KTI). An overview of (a) the theoretical determinants of occupational therapists’ and physical therapists’ intentions to use a variety of VR and other interactive technologies in practice and (b) their actual technology usage patterns **Design**: Quantitative **Participants**: Physical and occupational therapists (*n* = 11) **Data collection**: Survey	**VR technology and goal**: Nintendo Wii, WiiFit, Kinexct for XboX 360; in which the player is represented as an avatar. This system uses motion capture technology to allow full-body movements to control therapy-focused games developed with rehabilitation context in mind **Target group VR:** Rehabilitation patients **Setting**: Rehabilitation clinic	**Stage**: After implementation **Target group implementation**: Therapists **Objective**: N/S **Model:** Decomposed Theory of Planned Behavior + elements of Diffusion of Innovation Theory and Technology Acceptance Model (ADOPT-VR) **Strategies**: One-on-one mentoring sessions; **Outcomes**: Adoption and Acceptability
Bryant, Bluff, Barnett, Hemsley, Nguyen, Jacobs, Power, Baily, Stubbs, and Lucas, 2020; Australia [45]	**Goal**: Explore the views of professionals with expertise in health, rehabilitation, and VR technology, on the populations that might benefit from VR-based rehabilitation, and potential barriers and facilitators to their use of VR **Design**: Qualitative **Participants**: Health professionals (*n* = 9) and VR technologist (*n* = 1) **Data collection**: Focus group and interview	**VR technology and Goal**: Immersive VR using a head-mounted display (not specified further—VR technology is developed based on the insights of this study) **Target group VR**: Rehabilitation patients **Setting**: Rehabilitation clinic	**Stage**: Before implementation **Target group implementation**: Health professionals in rehabilitation **Objective**: N/S **Model:** N/S **Strategies**: N/A **Outcomes**: -
Cavenett, Baker, Waycott, Carrasco, Robertson, Vetere, and Hampson, 2018; Australia [36]	**Goal**: Explore factors that influence staff members when deploying new VR technology in residential aged care facilities **Design**: Qualitative **Participants**: Workers from Australian residential care facilities (*n* = 5) **Data collection**: Interviews	**VR technology and goal**: Commercial VR system with a headset, 2 3D cameras, 2 hand controllers. Aim is to let participants move things around in VR and stimulate physical activity **Target group VR**: Elderly at residential aged care facilities **Setting**: Residential aged care facilities	**Stage**: After implementation **Target group implementation:** Workers in residential aged care facilities **Objective**: N/S **Model:** N/S **Strategies**: N/S **Outcomes: -**
Chung, Robinson, Johnson, Dowling, Chee, Yücel, and Segrave, 2022; Australia [34]	**Goal:** Explore the perspectives of staff working in the private mental health sector around the use of therapeutic VR, including potential implementation barriers and facilitators **Design:** Qualitative **Participants:** Clinicians (*n* = 14) and managers (*n* = 5) of a major private mental health hospital **Data collection**: Interviews	**VR technology and goal:** A HTC Vive system with a wireless head-mounted display and handheld controllers was utilized. VR scenarios were designed for OCD treatment **Target group VR:** Patients with Obsessive–compulsive disorder (OCD) **Setting:** Private mental health hospital	**Stage:** Before implementation ***Use of VR***: VR only used as part of study, not in treatment **Target group implementation**: Clinicians **Objective:** N/S **Model:** Theory of Innovation Diffusion, CFIR and TDF **Strategies:** Treatment manuals; in-service training days; consultation opportunities with VR developers and early adopter services; Protocols to promote safe and ethical usage of VR **Outcomes**: -
Dahms, Stamm, and Muller-Werdan, 2019; Germany [54]	**Goal:** Determine the process-identifying needs of a VR training program for geriatric patients with chronic backpain **Design:** Qualitative **Participants:** Experts (*n* = 4) physiotherapists and psychotherapists in an executive position of a hospital and rehabilitation center **Data collection**: Interviews	**VR technology and goal:** ViRST: Personalized and adaptive VR based on immersive interaction sequences and gamification; sensor-based presentation of content with dynamic, adaptive and personalized storytelling for therapeutic recommendations through multimodal interaction with the content (Multimodal Pain Therapy) **Target group VR:** Chronic back pain patients **Setting:** Hospital and rehabilitation center	**Stage**: Before implementation **Target group implementation**: Experts who care for and have daily contact with geriatric, chronic back pain patients **Objective**: N/S **Model**: N/S **Strategies**: N/A **Outcomes**: -
Demers, Kong, and Levin, 2019; Canada [57]	**Goal:** Determine user satisfaction and safety of incorporating a low-cost virtual rehabilitation intervention as adjunctive therapeutic option for cognitive-motor upper limb rehabilitation in individuals with sub-acute stroke **Design:** Mixed-methods convergent parallel design: qualitative and quantitative cross-sectional **Participants:** Clinicians (*n* = 9) who are stroke program therapists and patients with a sub-acute stroke undergoing rehabilitation (*n* = 7) **Data collection**: Focus group, interviews and survey	**VR technology and goal:** Unity Pro software and Kineact II camera tracked arm, hand and trunk movements to interact with VR environment without a game controller. Projected on large screen. Participants played games sitting or standing with or without ambulatory aids. One smash blocks task and one shopper’s delight task (interactive grocery shopping) **Target group VR:** Stroke patients **Setting:** Rehabilitation clinic	**Stage**: Before implementation **Target group implementation**: N/S **Objective**: N/S **Model**: N/S **Strategies**: N/A **Outcomes**: Perceived usefulness, satisfaction
Demers, Nguyen, Austin Ong, Xin Luo, Thuraisingam, Rubino, Levin, Kaizer, and Archambault, 2019; Canada [35]	**Goal:** Understand the perspectives of clinicians regarding an exergaming program (VR) to supplement stroke rehabilitation care **Design:** Qualitative **Participants:** Occupational and physiotherapists (*n* = 10) working in the stroke program at a rehabilitation hospital **Data collection**: Interviews	**VR technology and goal:** Exergaming program in VR: Jintronix and Meditouch HandTutor to supplement stroke care and address therapeutic goals (e.g., improving upper limb function, sitting balance and endurance) **Target group VR:** Stroke patients **Setting:** Rehabilitation hospital	**Stage**: After implementation **Target group implementation**: Clinicians **Objective**: N/S **Model**: N/S **Strategies**: Obtaining a dedicated room for VR; approval from hospital administration; establishing the referral process; purchasing equipment; hiring personal for VR **Outcomes**: -
Easterlin, Berdahl, Rabizadeh, Spiegel, Agoratus, Hoover, and Dudovitz, 2020; USA [62]	**Goal:** Examining the acceptability of hypothetically using VR during an infusion appointment to help reduce medical trauma **Design:** Qualitative **Participants:** Patient-guardian dyads (*n* = 18) (pediatric IBD patients and parents) **Data collection**: Interviews	**VR technology and goal:** VR wear goggles to help reduce medical trauma (content not specified) **Target group VR:** Pediatric IBD patients **Setting:** Pediatric clinic	**Stage**: Before implementation **Target group implementation**: N/S **Objective**: N/S **Model**: N/S **Strategies**: N/A **Outcomes**: -
Ford, Mangegold, Randall, Aballay, and Duncan, 2018; USA [56]	**Goal:** Evaluate key stakeholder (i.e., patients, providers) perceptions of feasibility, acceptability, and effectiveness for the use of low-cost VR technology during routine burn care with adult patients **Design:** Quantitative cross sectional and qualitative **Participants:** patients (*n* = 10) within burn care and providers (*n* = 8) who delivered the burn care **Data collection**: Survey and interviews	**VR technology and goal:** VR is used as a distraction during burn care. An iPod Touch was used to deliver the VR videos: choice of 8 VR applications (Table Mountain sunset, reindeer race, scuba diving, exploring Amsterdam, roller coaster, playing soccer, swinging through a city, or riding motocross) **Target group VR:** Burn patients **Setting:** Burn care clinic	**Stage**: Before implementation **Target group implementation**: Care providers of burn patients **Objective**: N/S **Model**: N/S **Strategies**: N/A **Outcomes**: Acceptability and Feasibility
Glegg, Holsti, Stanton, Hanna, Velikonja, Ansley, Sartor, and Brum, 2017; Canada [43]	**Goal:** Evaluate the impact of knowledge translation (KT) on factors influencing virtual reality adoption and to identify support needs of therapists **Design:** Quantitative cross-sectional **Participants:** Physical, occupational, and rehabilitation therapists (*n* = 37) **Data collection**: Survey	**VR technology and goal:** A variety of VR and other interactive technology systems were already available to participants (not specified) **Target group VR:** Rehabilitation patients **Setting:** Brain injury rehabilitation centers	**Stage**: After implementation **Target group implementation**: Physical, occupational, and rehabilitation therapists **Objective**: Knowledge translation (KT) on factors influencing VR adoption and identify support needs of therapists **Model**: DTPB: Decomposed Theory of Planned Behavior (ADOPT-VR2 instrument) **Strategies**: Interactive education; clinical manual with goal setting, measuring client progress, developing client progress and evidence; Evidence synthesis; Sample goals; Overview of games and how to select goal-appropriate ones for clients; Information on isolating desired skills or grading VR activities for therapy **Outcomes**: Adoption and behavioral intention
Høeg, Scully, Bruun-Pedersen, and Serafin, 2020; Denmark [50]	**Goal:** Determine the circumstances in which physiotherapists and occupational therapists would decide to use VR as part of the therapy. Additionally, evaluate the challenges faced with the implementation, including pain points related to the use of VR **Design:** Qualitative **Participants:** Physiotherapists (*n* = 4) **Data collection**: Interviews and observations	**VR technology and goal:** VR-based treatment tool for biking-based rehabilitation: Oculus Rift Consumer Version headset. Software is a set of 4 unique, digitally generated virtual landscapes; measuring the angular velocity of the foot-pedals on the training bike. Stimulating movement **Target group VR:** Rehabilitation patients **Setting:** Outpatient health center	**Stage**: After implementation **Target group implementation**: Physiotherapists in outpatients health centers **Objective**: N/S **Model**: N/S **Strategies**: N/S **Outcomes**: -
Kramer, Jeffrey, Pyne, Timoty, Kimbrell, Savary, Jeffrey, Smith, and Jegley, 2010; USA [46]	**Goal:** Determine critical factors in the successful implementation of a VR intervention among veterans **Design:** Qualitative **Participants:** Clinicians (*n* = 18) from a Veterans Health Administration hospital **Data collection**: Focus groups	**VR technology and goal:** VR that offers an assessment method for OEF-OIF veterans by allowing for controlled immersion in a simulated combat environment while monitoring psychophysiological reactivity. The technology has also been used as an adjunct to exposure therapy and aims to improve PTSD symptoms among veterans **Target group VR:** Veterans **Setting:** Veterans Health Administration hospital (PTSD clinic, substance abuse treatment service residential program, and mental health clinic)	**Stage**: After implementation **Target group implementation**: Clinicians from a Veterans Health Administration hospital **Objective**: N/S **Model**: N/S **Strategies**: N/S **Outcomes**: -
Langlet, Odegi, Zandian, Nolstam, Södersten, and Bergh, 2021; Sweden [42]	**Goal:** Evaluate the feasibility and usability of an immersive virtual reality technology administered through an app for use of patients with eating disorders **Design:** Quantitative cross sectional **Participants:** Eating disorder personnel (*n* = 19) and information technology personnel (*n* = 5) **Data collection**: Usability tests and survey	**VR technology and goal:** Participants handled virtual food and utensils on an app using immersive virtual reality technology comprising a headset and two hand controllers. The challenge consisted of a meal type (meatballs, potatoes, sauce, and lingonberries) that is typically difficult for patients with anorexia nervosa to eat in real life. Participants were instructed, via visual feedback from the app, to eat at a healthy rate, which is also a challenge for patients **Target group VR:** Anorexia Nervosa patients **Setting:** Eating disorder clinic	**Stage**: Before implementation **Target group implementation**: Eating disorder clinic personnel **Objective**: N/S **Model**: N/S **Strategies**: N/A **Outcomes**: Feasibility (and usability)
Levac, Glegg, Pradhan, Foc, Espy, Chicklis, and Deutsch, 2019; USA [48]	**Goal:** Undertake a cross-country comparison of VR/AVG uptake to inform the content of educational interventions designed to promote implementation of these technologies into practice **Design:** Quantitative cross-sectional **Participants:** Physical- and occupational therapists in Canada and VS (*n* = 1490) **Data collection**: Survey	**VR technology and goal:** VR/AVG (active video gaming) in general healthcare (not specified) **Target group VR:** N/S **Setting:** General healthcare	**Stage**: After implementation **Target group implementation**: Physical- and occupational therapists **Objective**: N/S **Model**: Decomposed Theory of Planned Behavior (ADOPT-VR2 instrument) **Strategies**: N/S **Outcomes**: Uptake
Levac and Miller, 2013; Canada [13]	**Goal:** Explore observations and insights from a sample of physical therapists working with children with acquired brain injury regarding practical implications of using the Wii as a physical therapy intervention **Design:** Qualitative **Participants:** Physical therapists (*n* = 6) at a children’s rehabilitation center **Data collection**: Interviews	**VR technology and goal:** Wii virtual reality (VR) interactive video gaming console: movement-based games to target motor impairments in a variety of patient populations **Target group VR:** Variety of patient populations (sample: children with acquired brain injury) **Setting:** Clinical rehabilitation practice and at home	**Stage**: After implementation **Target group implementation**: Physical therapists at children’s rehabilitation center **Objective**: N/S **Model**: N/S **Strategies**: Making time to reflect and understand how to use VR; Spend time considering the potential and added value of VR into practice; Time and effort to set up and maintain system, find treatment location and maintenance issues; Getting familiar with the technology to instruct patients in its use **Outcomes**: -
Levac, Glegg, Sveistrup, Colquhoun, Miller, Finestone, DePaul, Harris, and Velikonja, 2016; USA [58]	**Goal:** (1) evaluate the impact of the intervention on therapists’ confidence related to VR knowledge and skills and perceptions of facilitators and barriers related to VR use; (2) assess the usability of the VR system; (3) obtain therapists’ perspectives about the KT intervention and VR use in practice; and finally, (4) measure the frequency of continued VR use following the KT intervention **Design:** Qualitative and quantitative cross-sectional **Participants:** Physical and occupational therapists (*n* = 11) **Data collection**: Focus groups and survey	**VR technology and goal:** Motion-capture technology enables players to view their mirror image in the virtual environment of the GestureTek Interactive Rehabilitation Exercise (IREX) software platform. Interaction with the virtual environment is through body movements to participate with games that address multiple upper extremity or full body movement goals, while motivating clients to participate **Target group VR:** Stroke rehabilitation patients **Setting:** Stroke rehabilitation units	**Stage**: After implementation **Target group implementation**: Physical- and occupational therapists **Objective**: The KT intervention designed to translate knowledge about use of the VR system to therapists in two stroke rehabilitation units **Model**: ADOPT-VR **Strategies**: E-learning modules (3 online modules provided foundational knowledge about clinical VR use); Hands-on learning (VR experience, training sessions, case scenarios); Experiential learning (use and reflect); Didactic reminders (weekly e-mails with “tips”for VR use); Mentorship (mentors to contact with questions or support needs) **Outcomes**: Uptake
Lindner, Miloff, Zetterlund, Reuterskiöld, Andersson, and Carlbring, 2019; Sweden [51]	**Goal:** Survey attitudes toward and familiarity with VR and VRET among practicing cognitive behavior therapists attending a conference **Design:** Quantitative cross-sectional **Participants:** Psychologists, psychiatrists, social workers, nurses, and counselors (*n* = 185) **Data collection**: Survey	**VR technology and goal:** Virtual reality exposure therapy (VRET) for fear and anxiety on VR headset **Target group VR:** Therapists **Setting:** Used during a conference (normally used during treatment)	**Stage**: Before implementation **Target group implementation**: N/S **Objective**: N/S **Model**: N/S **Strategies**: N/A **Outcomes**: -
Ma, Mor, Anderson, Baños, Botella, and Bouchard, 2021; Sweden [41]	**Goal:** Present an overview of current expert opinions on the use of virtual technologies in the treatment of anxiety and stress-related disorders **Design:** Quantitative cross-sectional **Participants:** Experts on VR and MR technology within psychotherapies (*n* = 14) **Data collection**: Survey	**VR technology and goal:** VR and MR technology use in treatment of anxiety and stress-related disorders (not specified) **Target group VR:** Patients with anxiety or stress-related disorders **Setting:** Clinics and within research projects	**Stage**: Before implementation **Target group implementation**: N/S **Objective**: N/S **Model**: N/S **Strategies**: N/A **Outcomes**: -
Nguyen, Ong, Luo, Thuraisingam, Rubino, Levin, Kaizer, and Archambault, 2019; Canada [37]	**Goal:** Identify the facilitators and barriers perceived by clinicians to using an Exergaming Room as adjunct to conventional therapy **Design:** Qualitative **Participants:** Clinicians (*n* = 10); physical therapists and occupational therapists **Data collection**: Interviews	**VR technology and goal:** The Exergames Room contains two systems. The Jintronix is a VR rehabilitation software and it elicits purposeful movements that can be done in sitting or standing. The Meditouch HandTutor allows the repetition of functional movements within a game context, while providing augmented motion biofeedback **Target group VR:** Rehabilitation patients **Setting:** The Exergames Room in a hospital	**Stage**: After implementation **Target group implementation**: Rehabilitation patients **Objective**: N/S **Model**: N/S **Strategies**: N/S **Outcomes**: -
Nwosu, Mills, Roughneen, Stanley, Chapman, and Rason, 2021; UK [52]	**Goal:** 1) explore the feasibility of implementing VR therapy, for patients and healthcare providers, in a hospital specialist inpatient palliative care unit and a hospice, and (2) to identify questions for organizations, to support VR adoption in palliative care **Design:** Quantitative cross-sectional **Participants:** Patients (*n* = 12) and healthcare providers (*n* = 3) **Data collection**: Survey	**VR technology and goal:** The Samsung Gear VR system was used in a hospital specialist palliative inpatient unit and a hospice. Patients and healthcare providers received VR distraction therapy **Target group VR:** Palliative patients **Setting:** Palliative inpatient unit	**Stage**: After implementation **Target group implementation**: Healthcare providers in palliative care units **Objective**: N/S **Model**: N/S **Strategies**: N/S **Outcomes**: Feasibility
Ogourtsova, Archambault, and Lamontagne, 2019; Canada [38]	**Goal:** Explore the barriers and facilitators perceived by clinicians in the use of virtual reality for hemineglect assessment; and to identify features of an optimal virtual assessment **Design:** Qualitative and quantitative cross-sectional **Participants:** Clinicians (*n* = 11) and research experts in the field (*n* = 3) **Data collection**: Focus groups, interviews, and survey	**VR technology and goal:** VR for post-stroke unilateral spatial neglect assessment: VR-based USN assessment that could be implemented and used by clinicians in the management of post-stroke unilateral spatial neglect **Target group VR:** Post-stroke patients **Setting:** Healthcare clinic	**Stage**: After implementation **Target group implementation**: Clinicians **Objective**: N/S **Model**: N/S **Strategies**: N/S **Outcomes**: -
Proffitt, Glegg, Levac, and Lange, 2019; USA [59]	**Goal:** Review four case examples from the authors’ collective experience of including end users in VR/AVG research to identify common benefits, challenges, and lessons learned **Design:** Quantitative cross-sectional and qualitative **Participants:** (1) therapists and clients with stroke; (2) OT students; (3) Clients and therapists; (4) Clients and therapists **Data collection**: Survey, interviews, observations, focus groups, and usability testing	**VR technology and goal:** Gesturetek IREX (interactive rehabilitation exercise system); Rapael SmartGlove; Adapted PlayStation2 controller; All VR systems related to rehabilitation **Target group VR:** Individuals with stroke, pro-bono clinic clients, adolescents and adults with hemiparesis, individuals with stroke/brain injury/amputations/older adults at risk for falls **Setting:** Inpatient stroke rehabilitation, pro-bono student-run outpatient clinic, home-based rehabilitation, outpatient clinic/hospital/home	**Stage**: After implementation **Target group implementation**: Therapists and clients **Objective**: N/S **Model**: N/S **Strategies**: N/S **Outcomes**: -
Rimer, Husby, and Solem, 2021; Norway [40]	**Goal:** Testing whether modern, wireless, commercially available VR equipment with controller-free hand tracking could induce and reduce discomfort using scenarios designed for fear of heights. Also, the study tested if clinicians’ attitudes toward using VR in therapy changed after trying it themselves **Design:** Quantitative cross-sectional **Participants:** Clinicians (*n* = 74) and psychology students (*n* = 54) with clinical experience **Data collection**: Survey	**VR technology and goal:** VRET software: The program utilized the VR hardware Oculus Quest, which is a wireless head-mounted display (HMD). It included a controller-free hand-tracking feature, which enabled the use of hands as an input method to control the program. The software consisted of two different scenarios, which will be referred to as the “Lift” and the “Plank.” **Target group VR:** Patients with anxiety/phobia treatment **Setting:** Private and public health clinics	**Stage**: After implementation **Target group implementation**: Clinicians from private and public health clinics **Objective**: N/S **Model**: N/S **Strategies**: N/S **Outcomes**: -
Sarkar, Lee, Nguyen, Lisker, and Lyles, 2021; USA [44]	**Goal:** Assess the readiness for VR in safety-net settings with a qualitative, theory-informed implementation science study **Design:** Qualitative **Participants:** Current VR users and non-users in safety-net health systems (*n* = 15) **Data collection**: Interviews	**VR technology and goal:** AppliedVR platform for pain treatment. A commercially available, previously validated VR technology platform (Not further specified) **Target group VR:** Chronic pain patients **Setting:** Safety-net sites—clinics	**Stage**: After implementation **Target group implementation**: Health care providers **Objective**: N/S **Model**: Consolidated Framework for Implementation Research (CFIR) **Strategies**: Specific orientation from staff in order to initiate VR use; Staff support for coaching and troubleshooting; Champions among clinicians who can share both evidence for VR and successful treatment experiences **Outcomes**: -
Stamou, Gracia-Palacios, and Botella, 2019; Spain [39]	**Goal:** Assess the level of feasibility, acceptance, and practical parameters of the combination of VR and traditional CBT for PND **Design:** Pilot study – quantitative cross-sectional and qualitative **Participants:** Patients (*n* = 6) with depression, anxiety, post-natal depression or recurrent depression **Data collection**: Survey and interviews	**VR technology and goal:** VR system where they were exposed to a series of virtual stressors, while at the same time, they were asked to tidy up the virtual house. VR stressors can be manipulated by the therapist in terms of intensity (from 0 to 6), frequency, duration, and applied individually or simultaneously. They are divided into three main categories and include amongst others: loud music, telephone ringing, newborn baby crying, toddler reaching for medication, power outage, fire in the kitchen, next door neighbors arguing, and next-door party **Target group VR:** Depression and anxiety-related patient groups **Setting:** General Practice clinics	**Stage**: Before implementation **Target group implementation**: N/S **Objective**: N/S **Model**: N/S **Strategies**: N/A **Outcomes**: Feasibility and Acceptability
Tennant, McGillivray, Youssef, McCarthy, and Clark, 2020; Australia [55]	**Goal:** 1. To evaluate the acceptability and feasibility of implementing an Immersive VR therapeutic intervention in an inpatient pediatric oncology setting, from the perspective of key stakeholders (i.e., oncology HCPs, patients, and parent healthcare providers); (2) to examine factors influencing VR adoption by HCPs, including barriers/facilitators to VR use with children who are seriously ill; (3) to explore user perspectives regarding the potential clinical utility of VR as an intervention to support psychological adjustment to hospitalization, including child VR content preferences **Design:** Quantitative **Participants:** Multidisciplinary oncology healthcare professionals (*n* = 30) and oncology inpatients (*n* = 90) **Data collection**: Survey	**VR technology and goal:** Immersive VR experiences were provided using a smartphone and VR headset and headphones. The intervention content involved original 360° video content. Participants viewed one of three virtual simulation experiences, including simulated travel to Australian national parks (i.e., nature experience), Australian zoos (i.e., animal experience), or global city tourist spots (i.e., travel experience). The goal is to support children’s needs during active cancer treatment, including to help regulate strong emotion, alleviate boredom, enhance mood, and provide a sense of escape from hospital, the experience of play, distraction from feared medical procedures, and physical symptom reduction **Target group VR:** Oncology inpatients (7–19 years) **Setting:** Children’s hospital and cancer center	**Stage**: Before implementation **Target group implementation**: Health care providers in children’s oncology care **Objective**: N/S **Model**: N/S **Strategies**: N/A **Outcomes**: Acceptability, adoption and feasibility
Üstel, Smith, Blajeski, Johnson, Butler, Nicola-Adkins et al., 2021; USA [47]	**Goal:** Understand peer specialist beliefs about potential barriers and facilitators influencing peer-delivered VR-JIT **Design:** Qualitative **Participants:** Peer specialists (*n* = 34) **Data collection**: Focus groups	**VR technology and goal:** Virtual Reality Job Interview Training (VR-JIT) which is a computerized job interview simulator delivered via the Internet. VR-JIT was designed to improve interview skills. VR-JIT enables trainees to review an e-learning curriculum about job interview strategies and tips; complete an online job application for a fictional company called “Wondersmart,” and then repeatedly practice interviews with a virtual hiring manager named “Molly Porter.” Trainees choose their responses from scripted options that range from highly effective to highly ineffective and then speak them aloud to “Molly Porter” using the website’s speech recognition function **Target group VR:** Individuals with serious illness **Setting:** Peer specialist workspaces	**Stage**: Before implementation **Target group implementation**: Peer specialists **Objective**: N/S **Model**: Consolidated Framework for Implementation Research (CFIR) **Strategies**: N/A **Outcomes**: Acceptability and Feasibility
Vincent, Eberts, Naik, Gulick, and O’Hayer, 2021, USA [53]	**Goal:** Explore the provider perception of the value of VR and identify barriers to Implementation among healthcare providers **Design:** Qualitative, cross-sectional **Participants:** Providers (*n* = 17) who have used VR as a therapeutic tool in the past year **Data collection**: Survey	**VR technology and goal:** VR as a treatment tool in psychiatry and pain management (not specified) **Target group VR:** Patients with psychiatric disorders or treated with pain management **Setting:** Community practice; medical clinic; research setting	**Stage**: After implementation **Target group implementation**: Healthcare providers **Objective**: N/S **Model**: N/S **Strategies**: Training about VR content in treatment and how to use VR (in person and online—about half a day); self-directed research **Outcomes**: -
